# Exploring the association between PITX2, third molars agenesis and sella turcica morphology

**DOI:** 10.1186/s13005-024-00414-4

**Published:** 2024-02-28

**Authors:** Erika Calvano Küchler, Jordanna de Melo Teixeira do Brasil, Isabela Ribeiro Madalena, Peter Proff, Flares Baratto-Filho, Mohammad Khursheed Alam, Angela Graciela Deliga Schroder, César Penazzo Lepri, Christian Kirschneck, Maria Angélica Hueb de Menezes-Oliveira

**Affiliations:** 1 Department of Orthodontics, Medical Faculty, University Hospital Bonn, Welschnonnenstraße 17, 53111 Bonn, Germany; 2 Department of Biomaterials, University of Uberaba, Uberaba, Minas Gerais Brazil 38.055-500 Nenê Sabino, 1801,; 3 Department of Orthodontics, University of Regensburg, Regensburg, Germany Franz-Josef-Strauß-Allee, 11, 93053; 4 School of Dentistry, Tuiuti University from Parana, Curitiba, Paraná Brazil Padre Ladislau Kula, 395, 82010-210; 5 Department of Dentistry, University of Joinville Region, Joinville, Santa Catarina Brazil Paulo Malschitzki , 89219-710; 6 College of Dentistry, Jouf University, Al-Jouf, Saudi Arabia

**Keywords:** Sella turcica, Tooth agenesis, Gene

## Abstract

**Objectives:**

PITX2 is required for mammalian development and single nucleotide polymorphisms (SNPs) in this gene could be involved in dental agenesis and sella turcica patterns. Thus, the present study evaluated the association between SNPs in *PITX2*, third molars agenesis and sella turcica phenotypes.

**Materials and methods:**

The sample consisted of healthy orthodontic German patients with lateral cephalometric radiographs with clearly visualization of the sella turcica, and dental orthopantomograms. The morphological variations of the sella turcica were evaluated using the lateral cephalograms, while third molar agenesis was evaluated using orthopantomograms. DNA isolated from buccal cells was used for genotyping three SNPs in *PITX2* (rs3796902, rs1947187, and rs2595110). The analyzes were performed using a significance of 5%. There was no association between third molar agenesis and sella turcica phenotypes (*p* > 0.05). SNPs in *PITX2* were also not associated with third molars agenesis (*p* > 0.05).

**Results:**

SNPs in *PITX2* were associated with sella turcica phenotypes. The rs3796902 was associated with hypertrophic posterior clinoid process (*p* = 0.013). The rs1947187 and rs2595110 were associated with sella turcica bridge type A (*p* = 0.013 and *p* = 0.011, respectively for genotype distribution). Patients that carry the genotypes GG-CC-AG (rs3796902- rs1947187- rs2595110) had 7.2 higher chance to present sella turcica bridge type A (*p* = 0.002; Odds ratio = 7.2, Confidence interval 95% 2.04–27.04).

**Conclusions:**

Third molar agenesis was not associated with SNPs in *PITX2* and sella turcica phenotypes. SNPs in *PITX2* may have an important role in sella turcica pattern.

## Introduction

Third molars are unique dental elements that exhibit a remarkable long developmental time in humans. They are the last teeth to erupt into the oral cavity [1], and are commonly affected by many developmental dental alterations, especially dental agenesis [2, 3]. Dental agenesis is a developmental dental anomaly that affects the number of teeth, leading to the congenital absence of one or more teeth. Third molars are the most commonly congenitally missing teeth in the permanent dentition [4]. A systematic review with meta-analysis estimated that the worldwide frequency of third molar agenesis is 22.6%.^5^.

The sella turcica is an important anatomical structure located on the intracranial surface of the body of the sphenoid and consists of a central pituitary fossa [6]. Two anterior and two posterior clinoid processes project over the pituitary fossa. Fusion of the posterior and anterior clinoid processes is known as a sella turcica bridge. Calcification of the interclinoid ligament of the sella turcica is also a common phenotype observed in the general population [7]. 

In the past two decades, some original studies [7–12] and systematic reviews [13, 14] have been investigating the association between dental agenesis of permanent teeth and sella turcica phenotypes, suggesting that patients with dental agenesis have a higher chance to present morphological variations of the sella turcica. The development and the formation of sella turcica and teeth share the involvement of neural crest cells. The anterior part of sella turcica develops mainly from neural crest cells, while progenitor cells of dental epithelium differentiate by sequential and reciprocal interaction with the neural crest-derived mesenchymal tissue [15]. Therefore, the formation of both structures, teeth and sella turcica, might share some genetic factors in common. A recent phenotype-genotype study investigated the association between sella turcica phenotype and single nucleotide polymorphisms (SNPs) located in the members of the wingless-type MMTV integration site family (WNT), the study found an association between *WNT10A* with the calcification phenotypes of the sella turcica [16]. Interestingly, *WNT10A* have been also associated with dental agenesis [17, 18], supporting the hypothesis that the connection between sella turcica and dental alterations comes from a common genetic background. Dental alterations and sella turcica patters are polygenic conditions, in which several genes play a role on the development of these structures. *PITX2* is also a candidate gene that could be involved in dental agenesis and sella turcica phenotypes.

In the current study we hypothesize that *PITX2* have a pleiotropic effect acting on sella turcica morphogenesis and dental development. Thus, in this study we investigated the association between third molars agenesis and sella turcica phenotypes. We also explored the role of SNPs in *PITX2* on dental agenesis and sella turcica pattern investigating if *PITX2* could be a common genetic factor for both traits.

## Materials and methods

The approval for this research was obtained by the local Ethics Committee from the University of Regensburg (#19-1549-101). Informed consent was obtained from all included patients and the assent was also obtained from any participant younger than 18 years during dental appointment. The guideline STREGA (Strengthening the Reporting of Genetic Association) was followed for this cross-sectional study [19]. 

The study sample was composed by lateral cephalometric radiographs and dental orthopantomograms from patients, aged 12 to 35 years old, undergoing orthodontic treatment at the University of Regensburg and private orthodontic practices in Regensburg, Germany. In cases of patients that started the orthodontic treatment before 12 years old, they were only included if follow up radiographs (at 12 years old or older) were present in the dental records to allow the confirmation of the congenital absence of the third molars. All radiographs were made for orthodontic treatment purposes. The patients included in this study were recruited from 2020 to 2021.

The exclusion criteria were incomplete records, the presence of syndromes, oral clefts, unclear visualization (blurred images) of the sella turcica, other types of tooth agenesis besides third molar agenesis, and previous extraction of third molars. To minimize genetic variance, only patients with a Central-European ancestry (at maximum one grandparent not from Central Europe), and one patient per family were included.

### Dental orthopantomograms analysis

All dental orthopantomograms were digital panoramic radiographs that were examined in a dark room using the same protocol. In all cases third molar agenesis were clearly evident from the panoramic radiographs alone using a method previously reported [20]. In case of doubt, a follow-up orthopantomograms of the same patient in the course of orthodontic treatment was examined to confirm the agenesis diagnosis, as reported in Herrmann et al. [21] If tooth agenesis could not be confirmed, the patient was excluded. Each panoramic orthopantomograms was evaluated by one dentist. For intra-examiner reliability and inter-examiner reliability, 10% of the orthopantomograms were randomly chosen and the investigations were conducted twice in a 2-weeks interval. The Kappa statistics showed perfect agreement to both tests for third molar agenesis (Kappa for all third molar agenesis was 1).

Tooth agenesis was defined based on the age of patients and when initial third molar should be visible in the radiographs [20]. Patients were considered with third molar agenesis when at least one third molar was. Patients with all 32 permanent teeth were considered as non-tooth agenesis patients.

### Cephalometric analysis

All the cephalometric radiographs were also taken in Frankfort horizontal plane parallel to the floor in a rigid cephalostat.

**Fig. 1:**
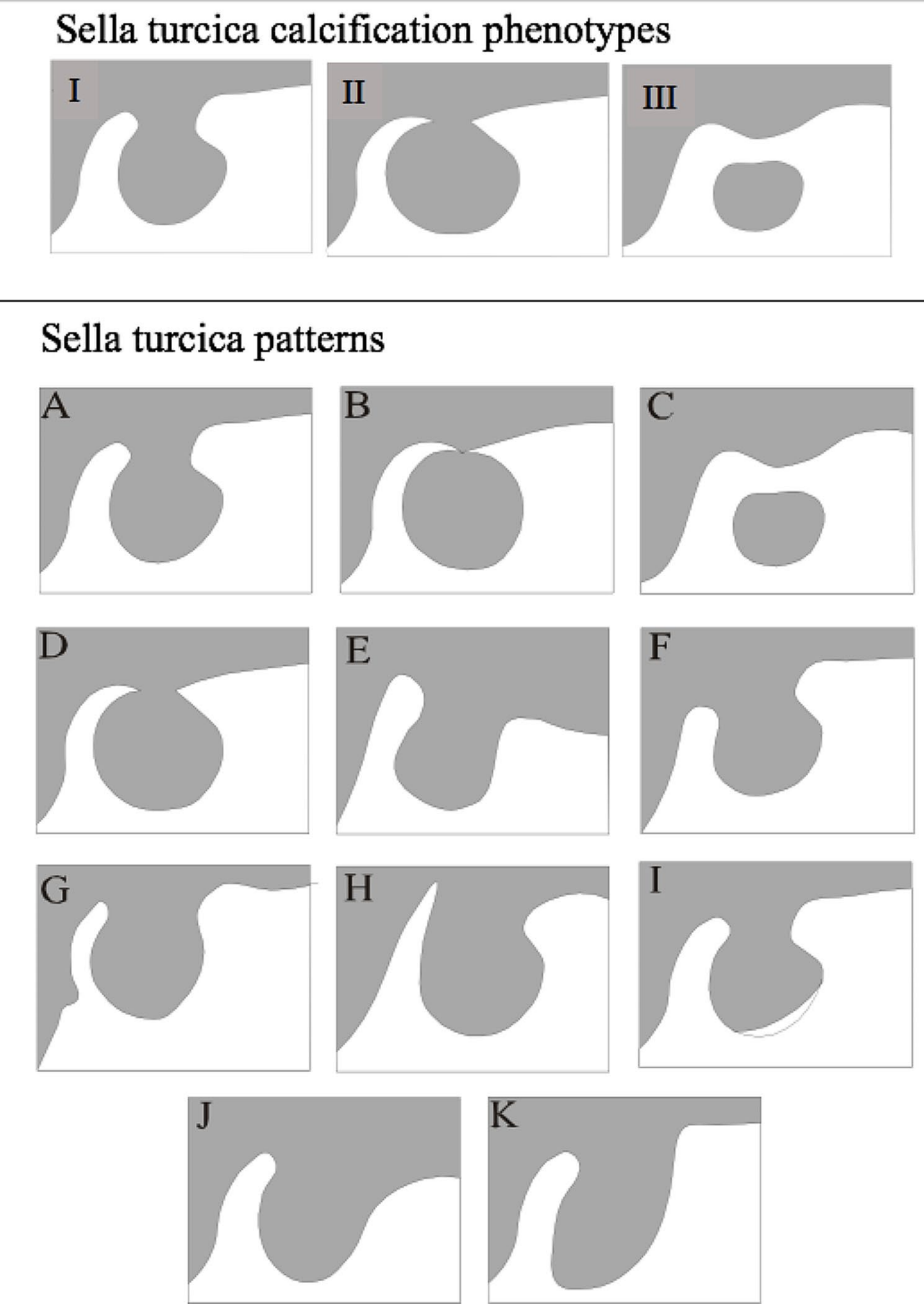
Sella turcica classifications Sella turcica calcification phenotypes (**I**- calcification; **II**- partially calcified; **III**- completely calcified). Sella turcica patterns (**A**- normal sella turcica; **B**- sella turcica bridge type B- extension of the clinoid process; **C**- sella turcica bridge type A- ribbon-like fusion; **D**- incomplete bridge; **E**- hypertrophic posterior clinoid process; **F**- hypotrophic posterior clinoid process; **G**- irregularity (notching) in the posterior part of the sella turcica; **H**- pyramidal shape of the dorsum sella; **I**- double contour of the floor; **J**- oblique anterior wall; **K**- oblique contour of the floor)

The sella turcica calcification was evaluated in three aspects: (1) no calcification, (2) partial calcification and (3) complete calcification of the interclinoid ligament [7, 8, 11, 12] as shown in the Fig. 1. Figure 1 also shows the sella turcica patterns, which was evaluated according to the protocol described by Kucia et al. [22].

All cephalometric radiographs were examined by only one trained and calibrated examiner. For inter-examiner reliability, 10% of the sample were randomly chosen for a second analysis in a 2-week interval. The Kappa statistics showed perfect agreement (Kappa = 1) for sella calcification and for sella turcica pattern.

**Fig. 2:**
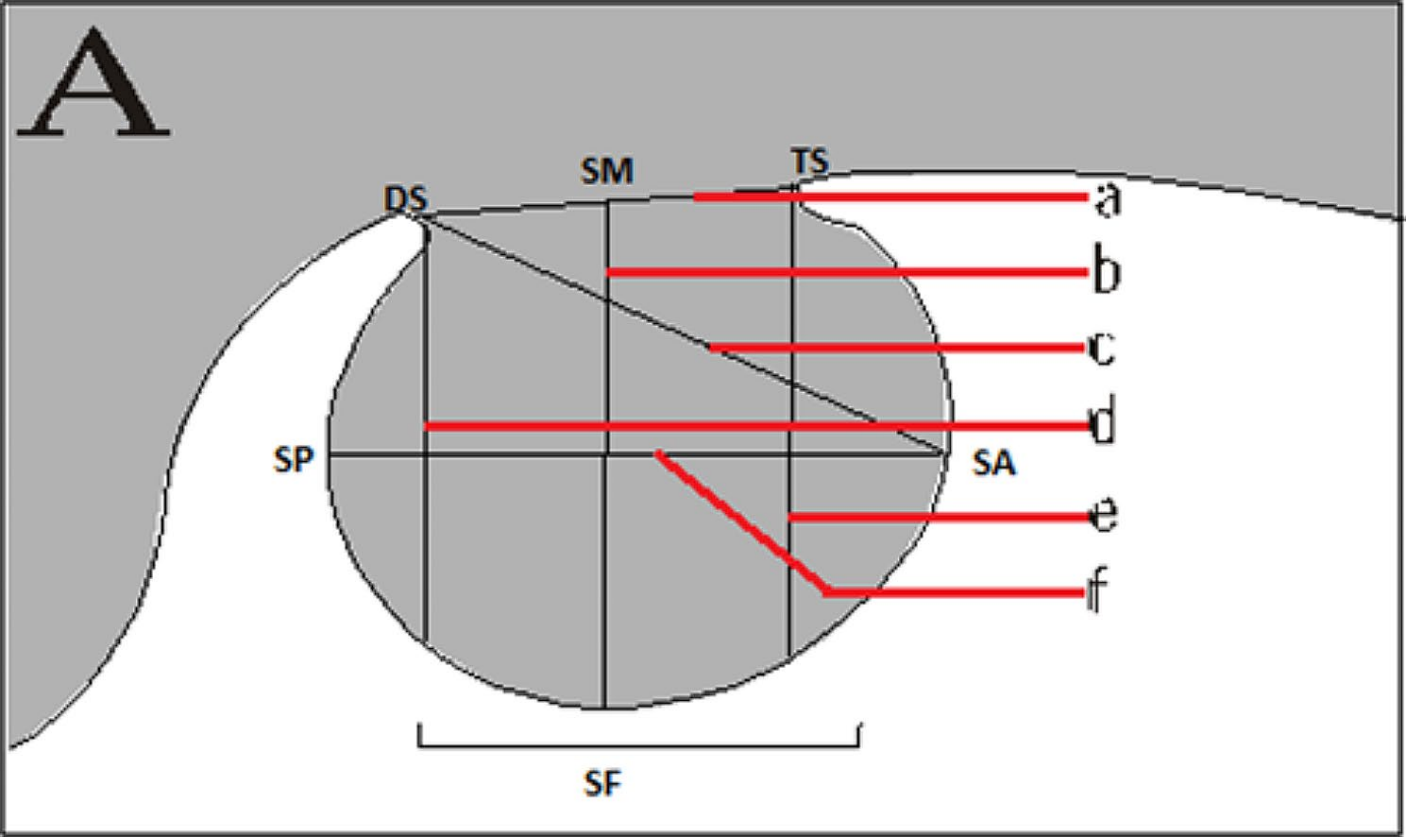
Sella turcica landmarkers and linear measurements. **a**: length; **b**: depth; **c**: diameter; **d**: anterior height; **e**: posterior height; **f**: width SF means sella floor

The landmarkers were established for each radiograph using the Frankfurt plane (FH) as the horizontal reference direction. The total of six points defined the measurements performed (Fig. 2). The following linear and area measurements were calculated sella measurements:

1. Sella length: Distance between the dorsum sellae (DS) and the tuberculum sella (TS).
2. Sella depth: Distance between midpoint clinoid process DS and TS to SF, perpendicular to FH.
3. Sella diameter: Distance between the tuberculum sella to the most posterior point on the inner wall of the pituitary fossa.
4. Sella height anterior: The vertical distance, as measured perpendicular to the FH plane, from Tuberculum sella (TS) to the sella floor (SF).
5. Sella height posterior: The vertical distance, as measured perpendicular to the FH plane, from DS to the sella floor (SF).

Sella width: Distance between of the point most posterior (SP) and the point most anterior (SA) perpendicular to the Frankfurt plane (FH).

6. Sella area: the area included by the outline of the sella and capped by a line joining TS- SA – SF – SP - DS.

All measurements were performed after adjusting for the magnification of the radiographs in a room with dimmed light. The ImageJ software version 1.53a (Rasband, W.S., ImageJ, U.S. National Institutes of Health, Bethesda, MD, USA) was used. To calculate the error of the method, 20 radiographs were randomly selected and reanalyzed. The intraclass correlation coefficient (ICC) was used to evaluate the inter-examiner agreement, and the ICC ranged from 0.915 to 0.998.

### DNA extraction and genotyping

The intronic SNPs rs3796902, rs1947187, and rs2595110 in PITX2 were screened from the dbSNP database (http://www.ncbi.nlm.nih.gov/snp/) based on their MAF (minor allele frequency) (≥ 10% in the global population).

The genomic DNA of each included patient was used for the genotyping analysis. The DNA was isolated from epithelial cells collected with cytobrushes using extraction solution (Tris-HCl 10 mmol/L, pH 7.8; EDTA 5 mmol/L; SDS 0.5%, 1 mL) and proteinase K (100 ng/mL) and ammonium acetate to remove non-digested proteins. The DNA was precipitated with isopropanol and resuspended, and later quantified by spectrophotometry (Nanodrop 1000; Thermo Scientific, Wilmington, DE, USA) [23]. All 3 SNPs were blindly genotyped using real-time Polymerase chain reaction (real time-PCR) in the Mastercycler® ep realplex-S thermocycler (Eppendorf AG, Hamburg, Germany). The TaqMan technology, which uses extremely sensitive allele-specific probes (VIC™ and FAM™ dyes were used for the alleles), was used in this study. One negative control template (omitting the DNA) was used in each reaction plate. Additionally, 10% of the samples were randomly selected for repeated analysis (presented 100% concordance). DNA samples that failed to be genotyped were considered missing data and was excluded from the statistical analysis.

### Statistical analysis

For the sample size estimation, the calculation with an effect size *w =* 0.23, Degrees of freedom = 1, α = 0.05, and β = 0.80 predicted a minimum of 143 individuals [21].

The Hardy-Weinberg Equilibrium was calculated for each SNP Chi-square test (wpcalc.com/en/equilibrium-hardy-weinberg).

Epi Info was used for statistical analysis. Sella turcica phenotypes were compared among third molar agenesis and controls groups. Also, SNPs in *PITX2* were analyzed according to tooth agenesis and sella turcica phenotypes (regardless tooth agenesis condition). Chi-square test or Fisher’s exact test were used to compare genotype and allele distribution according to the phenotypes. ANOVA with Tukey’s multiple-comparison test and t test were also used to compare the means among groups and genotypes. SNP-SNP interaction was also tested. The significance level was set as 5% (*p* < 0.05) for all comparisons.

## Results

A total of 163 patients were included. Fourth-one (20 males and 21 females) patients presented third molar agenesis and 122 (62 males and 60 females) were patients without tooth agenesis (controls). Gender distribution among tooth agenesis groups was not statistically significant different (*p* = 0.821).

Calcification sella turcica was more common phenotype, 107 patients presented partially phenotype sella, 20 presented completely calcified sella and 36 presented no calcification. Sella turcica calcification was not statistically significant different between genders (*p* = 0.535).

Sella turcica bridge type B was the most common type (*n* = 45), followed by the normal sella turcica (*n* = 36). Sella turcica formats were not statistically significant different between genders (*p* = 0.653).

Table 1 shows the sella turcica phenotypes frequency and distribution among third molar agenesis and control groups. There were no statistical differences among the groups (*p* > 0.05 for all comparisons).

**Table 1:** Sella turcica phenotypes among third molar agenesis and controls

Parameter	Third molar agenesis (*N* = 41)	Control (*N* = 122)	*p*-value
	Mean (SD)	Mean (SD)	
Length (mm)	8.58 (1.66)	8.79 (1.94)	0.506
Depth (mm)	8.28 (1.14)	8.16 (1.30)	0.582
Posterior height (mm)	8.46 (1.32)	8.24 (1.50)	0.391
Anterior height (mm)	8.10 (1.48)	8.07 (1.56)	0.909
Width (mm)	8.65 (1.31)	8.68 (1.53)	0.900
Diameter (mm)	10.67 (1.36)	10.96 (1.93)	0.291
Area (mm [2])	61.12 (13.55)	62.33 (16.93)	0.645
**Degree of calcification**	**N (%)**	**N (%)**	
No calcification	7 (17.1)	29 (23.8)	0.627
Partially calcified	28 (68.3)	79 (64.7)	
Completely calcified	6 (14.6)	14 (11.5)	
**Format of Sella turcica**	**N (%)**	**N (%)**	
Normal ST	8 (19.5)	30 (24.6)	0.657
ST bridge type A	6 (14.6)	13 (10.6)	
ST bridge type B	12 (29.3)	33 (27.0)	
Incomplete bridge	2 (4.9)	10 (8.2)	
Hypertrophic posterior clinoid process	8 (19.5)	21 (17.2)	
Hypotrophic posterior clinoid process	0 (0)	1 (0.8)	
Irregularity in the posterior part of the ST	2 (4.9)	1 (0.8)	
Pyramidal shape of the dorsum of the ST	0 (0)	2 (1.6)	
Double contour of the floor	0 (0)	1 (0.8)	
Oblique anterior wall	1 (2.4)	8 (6.6)	
Oblique contour of the floor	2 (1.6)	2 (4.8)	

Note: t test was used. SD means standard deviation

The association of the SNPs rs3796902, rs1947187 and rs2595110 with third molar agenesis is presented in the Table 2. The genotype distribution was not statistically significant different among the groups (*p* > 0.05).

**Table 2:** Genotype frequency distribution between third molar agenesis group and control group and comparison

SNP	rs3796902			*p*-value	
**Genotypes**	**AA**	**AG**	**GG**	**Genotype**	**Allele**
Control	4 (3.6)	38 (34.2)	69 (61.2)	0.459	0.356
Third molar agenesis	0 (0)	11 (31.4)	24 (68.6)		
**SNP**	**rs1947187**			**Genotype**	**Allele**
**Genotypes**	**CC**	**CT**	**TT**		
Control	86 (78.9)	19 (17.4)	4 (3.7)	0.681	0.618
Third molar agenesis	25 (73.9)	8 (23.5)	1(2.9)		
**SNP**	**rs2595110**			**Genotype**	**Allele**
**Genotypes**	**AA**	**AG**	**GG**		
Control	40 (35.4)	55 (48.7)	18 (15.9)	0.950	0.998
Third molar agenesis	13 (36.1)	17 (47.2)	6 (16.7)		

Note: Chi-square was used

Sella turcica linear and area measurements among genotypes are presented in the Table 3. Means differences were not observed among genotypes (*p* > 0.05).

**Table 3:** Means comparisons of Sella Turcica’s parameters according to the genotypes

SNP	rs3796902				rs1947187				rs2595110			
Parameters	AA	AG	GG	*p*-value	CC	CT	TT	*p*-value	AA	AG	GG	*p*-value
**Length (mm)**	83.75 (18.21)	86.85 (18.44)	86.20 (17.25)	0.936	86.66 (17.19)	86.16 (16.70)	79.58 (19.55)	0.665	87.45 (18.57)	86.75 (16.92)	82.25 (16.42)	0.458
**Depth (mm)**	80.43 (8.15)	83.13 (12.39)	82.01 (12.00)	0.826	82.22 (12.17)	82.72 (11.73)	80.40 (9.75)	0.922	82.23 (13.49)	82.71 (11.60)	82.44 (10.55)	0.976
**Posterior height (mm)**	83.62 (8.56)	85.58 (13.89)	82.96 (14.21)	0.573	83.46 (14.71)	86.75 (11.81)	81.71 (8.67)	0.517	81.10 (16.56)	80.32 (13.73)	82.76 (16.05)	0.745
**Anterior height (mm)**	76.03 (10.44)	81.22 (15.07)	80.96 (15.28)	0.803	80.98 (15.41)	78.99 (14.51)	79.24 (13.35)	0.814	81.10 (16.56)	80.32 (13.73)	82.76 (16.05)	0.873
**Width (mm)**	83.99 (19.34)	87.16 (13.29)	86.45 (14.09)	0.892	86.13 (13.16)	89.12 (14.34)	87.01 (18.74)	0.591	86.48 (14.82)	87.07 (12.80)	86.68 (14.92)	0.971
**Diameter (mm)**	10.05 (12.59)	10.58 (14.29)	10.79 (16.62)	0.523	10.72 (16.05)	10.68 (12.63)	10.63 (22.49)	0.984	10.62 (18.18)	10.84 (14.04)	10.45 (14.10)	0.519
**Area (mm** ^**2**)^	56.43 (13.54)	62.64 (14.54)	60.94 (14.99)	0.643	60.70 (14.32)	63.83 (14.52)	57.10 (15.09)	0.487	62.01 (16.19)	61.63 (14.18)	60 0.30 (13.12)	0.893

Note: ANOVA and Tukey’s post hoc test were used

Table 4 shows the genotype distribution among degree of calcification phenotypes are presented in the Table 4. The genotype distribution was not associated with the degree of calcification (*p* > 0.05). Allele distribution was also not associated with the degree of calcification (*p* > 0.05).

**Table 4:** Genotype frequency distribution and comparison between degree of calcification

SNP	rs3796902				rs1947187				rs2595110			
Degree of calcification	AA	AG	GG	*p*-value	CC	CT	TT	*p*-value	AA	AG	GG	*p*-value
No calcification	0 (0.0)	12 (37.5)	20 (62.5)	0.564	24 (77.4)	7 (22.6)	0 (0.0)	0.360	10 (31.3)	14 (43.7)	8 (25.0)	0.207
Partially calcified	3 (3.2)	3 (34.8)	59 (62.1)		70 (75.3)	19 (20.4)	4 (4.3)		39 (39.8)	45 (45.9)	14 (14.3)	
Completely calcified	1 (5.3)	4 (21.0)	14 (73.7)		17 (89.5)	1 (5.3)	1 (5.3)		4 (21.0)	13 (68.4)	2 (10.6)	

Note: Chi-square was used

Table 5 shows genotype and allele distribution among sella turcica patterns. The rs3796902 was associated with hypertrophic posterior clinoid process (*p* = 0.039 for genotype distribution and *p* = 0.050 for allele distribution). The rs1947187 and rs2595110 were associated with sella turcica bridge type A (*p* = 0.013 and *p* = 0.011 respectively for genotype distribution). Patients that carry the genotypes GG-CC-AG (rs3796902- rs1947187- rs2595110) had 7.2 higher chance to present sella turcica bridge type A (*p* = 0.002; Odds ratio = 7.2, Confidence interval 95% 2.04–27.04).

**Table 5:** Genotype frequency distribution and comparison between degree of calcification

SNP	rs3796902				rs1947187					rs2595110						
Patterns	AA	AG	GG	*p*-value Genotype	*p*-value Allele	CC	CT	TT	*p*-value Genotype	*p*-value Allele	AA	AG	GG	*p*-value Genotype		*p*-value Allele
Normal sella turcica	1 (2.9)	13 (38.2)	20 (58.9)	Reference		25 (75.8)	6 (18.2)	2 (6.0)	Reference		14 (41.2)	11 (32.3)	9 (26.5)	Reference		
ST bridge type B	2 (5.1)	16 (41.0)	21 (53.9)	0.849	0.621	26 (68.4)	10 (26.3)	2 (5.3)	0.715	0.677	17 (42.5)	18 (45.0)	5 (12.5)	0.265		0.340
ST bridge type A	0 (0.0)	4 (25.0)	12 (75.5)	0.478	0.290	18 (100)	0 (0.0)	0 (0.0)	0.075	0.013*	3 (17.6)	13 (76.5)	1 (5.9)	0.011*		0.887
Incomplete bridge	0 (0.0)	6 (66.7)	3 (33.3)	0.296	0.198	6 (66.7)	3 (33.3)	0 (0.0)	0.501	0.482	3 (30.0)	4 (40.0)	3 (30.0)	0.811		0.892
Hypertrophic posterior clinoid process	1 (3.5)	3 (10.3)	25 (86.2)	0.039*	0.050*	22 (78.6)	5 (17.9)	1 (3.6)	0.891	0.782	10 (34.5)	16 (55.2)	3 (10.3)	0.121		0.318
Hypotropic posterior clinoid process	0 (0.0)	1 (100)	0 (0.0)	0.462	0.562	1 (100)	0 (0.0)	0 (0.0)	0.853	0.872	0 (0.0)	0 (0.0)	1 (100)	0.271		0.922
Irregularity in the posterior part of the ST	1 (20.0)	1 (20.0)	3 (60.0)	0.237	0.872	3 (100)	0 (0.0)	0 (0.0)	0.839	0.792	0 (0.0)	2 (66.7)	1 (33.3)	0.331		0.832
Pyramidal shape of the dorsum sella	0 (0.0)	2 (100)	0 (0.0)	0.227	0.235	0 (0.0)	2 (100)	0 (0.0)	0.028	0.133	1 (50.0)	1 (50.0)	0 (0.0)	0.592		0.899
Double contour of the floor	0 (0.0)	1 (100)	0 (0.0)	0.462	0.562	1 (100)	0 (0.0)	0 (0.0)	0.853	0.872	1 (100)	0 (0.0)	0 (0.0)	0.503		0.628
Oblique anterior wall	0 (0.0)	1 (12.5)	7 (87.5)	0.308	0.285	7 (100)	0 (0.0)	0 (0.0)	0.346	0.588	2 (25.0)	5 (62.5)	1 (12.5)	0.285		0.632
Oblique contour of the floor	0 (0.0)	2 (50.0)	2 (50.0)	0.509	0.289	2 (66.7)	1 (33.3)	0 (0.0)	0.792	0.690	2 (50.0)	2 (50.0)	0 (0.0)	0.484		0.559

Note: Chi-square was used. * Means statistical significance difference

## Discussion

The association between sella turcica morphology and developmental dental anomalies has been an area of interest for some research groups,[7–9, 11−13, 24–26] and many of them suggested the existence of an association between sella turcica morphology and tooth agenesis [7–9, 11, 12]. In our study, third molar agenesis was not associated with sella turcica morphology, however, it is important to emphasize that third molar agenesis has several unique characteristics, and the previous studies that observed the association between tooth agenesis and sella turcica morphology investigated other congenitally missing teeth than third molars. The previous studies that found an association, excluded third molars from the analysis [7–9, 11]. Third molars are the last teeth to develop and to erupt into the oral cavity (eruption time range from 17 to 24 years) [1]. Also, third molars are the most common congenitally missing and the prevalence is approximately 21.6% in the European population [5], in contrast to other type of teeth, that the prevalence in Europe is 4.6% in males and 6.3% in females [27]. 

PITX2 is one of the earliest transcription factors observed during dental development. It activates genes important for tooth development within the dental epithelium during dental development [28]. PITX2 is also regulated by the WNT/β-catenin pathway and functions in the pathway [29–31]. WNT gene is well known involved in tooth agenesis [17, 18]. In our sample the investigated SNPs in *PITX2 *were not associated with third molar agenesis. The function of PITX2 is highly conserved during vertebrate development. Studies using animal models support the importance of PITX2 in tooth development [32, 33]. Therefore, it is possible that these SNPs could be associated with other types of missing teeth.

SNPs in *PITX2* were associated with some sella turcica patterns. The SNPs rs2595110 and rs1947187 were associated with sella turcica bridge type A (in genotypic and/or allelic models). The SNP-SNP interaction also showed that patients that carry the genotypes GG-CC-AG (rs3796902- rs1947187- rs2595110) had 7.2 higher chance to present sella turcica bridge type A. Sella bridging is a frequent morphological variation of the sella turcica. Excessive ossification of the ligaments between the anterior and posterior clinoid processes of the sphenoid lead to the development of the bridge [34]. Interestingly, some authors reported that sella turcica bridging is likely to complement the diagnostic parameters to predict the susceptibility of some dental alterations [35], and was also associated with skeletal malocclusion, in which skeletal class III malocclusion presented more sella bridging [34, 36]. 

Posterior to the sella are the posterior clinoid processes. The rs3796902 was associated with hypertrophic posterior clinoid process, in which patients carrying GG genotype had higher chance to present this sella turcica pattern. The anterior and posterior walls of the sella turcica have different developmental origin, in which the anterior wall develops from the neural crest cells and the posterior wall develops from paraxial mesoderm under the direct influence of notochord [37]. 

The sella turcica is an anatomical structure readily recognized on lateral cephalometric radiographs and highly important for cephalometric analysis due to its central landmark [38]. In this study we used cephalometric analysis to access sella turcica morphology. Cephalograms are used in orthodontics to assess the craniofacial morphology. Although pretreatment lateral cephalograms are mainly used for evaluation of skeletal and dental patterns, they are skull radiographs and contain other diagnostic information about the upper cervical spine, face and skull, allowing to explore the sella turcica morphology, even though it has a limitation of a two-dimensional exam, that present higher risk of detecting errors because of the low contrast resolution and superposition of the overlapping structure anatomical structures. On the other hand, studies evaluating sella turcica morphology using three-dimensional exam (computed tomography and cone-beam computed tomography) have more accurate and detailed visualization of the anatomical variation and landmarkers of the sella turcica and therefore could produce a more exact characterization of the sella area [39]. 

This is the first evidence that SNPs in *PITX2* could be involved in the sella turcica pattern. Interestingly, there is evidence that PITX2 interact with WNT pathway [40]. In our previous study, the SNP rs10177996 in *WNT10A* was associated with sella turcica calcification, but not with sella turcica patterns [17]. 

## Conclusion

Third molar agenesis was not associated with sella turcica phenotypes and SNPs in *PITX2*. SNPs in *PITX2* may have an important role in sella turcica pattern.

## Data Availability

No datasets were generated or analysed during the current study.
